# International Frontal Sinus Anatomy Classification (IFAC): evaluation of frontoethmoidal cells prevalence in a Brazilian population

**DOI:** 10.1016/j.bjorl.2023.101309

**Published:** 2023-08-23

**Authors:** Ruth Ellen Fernandes de Castro Dantas Braz, Mariana Dalbo Contrera Toro, Emerson Taro Inoue Sakuma, Vinicius Silles Brandão Machado, Eulalia Sakano

**Affiliations:** a Universidade Estadual de Campinas, Faculdade de Ciências Médicas, Departamento de Otorrinolaringologia, Campinas, SP, Brazil; b Universidade Estadual de Campinas, Faculdade de Ciências Médicas, Departamento de Radiologia, Campinas, SP, Brazil

**Keywords:** Frontal sinus, Ethmoid sinus, Tomography, Anatomy

## Abstract

**Objectives:**

The aim of this study was to evaluate the prevalence of the frontal cell variants according to International Frontal Sinus Anatomy Classification (IFAC), in the population of a Brazilian tertiary hospital, and analyze the reliability of the classification between observers.

**Methods:**

A cross-sectional study in the Hospital de Clínicas of the State University of Campinas, Brazil. One hundred and three Computed Tomography's (CTs) were evaluated by radiologists and otorhinolaryngologist to estimate the prevalence of frontoethmoidal cells according to the IFAC. Intraclass Correlation Coefficient (ICC) among examinators was used to evaluate reliability of this findings.

**Results:**

103 CT scans, totaling 206 sides, were evaluated independently. The agger nasi cell was the most prevalent, present in 95.63% of cases, 37.86% of the exams contained supra agger cells, frontal supra agger cell showed prevalence 37.37%; suprabular cell was present in 77.18% of the sides. As for the frontal suprabular cell, the prevalence was 30.09%, the supraorbital ethmoid cell was present in 32.03%, and frontal septal cell had a 33.49% prevalence. The ICC among the evaluators was classified as “good reliability” or “excellent reliability” for all cells.

**Conclusion:**

This study describes the frontal cell prevalence among a population in tertiary Brazilian hospital, using the IFAC. This classification had a high ICC.

**Level of evidence:**

Level 2: Individual cross-sectional study with consistently applied reference standard and blinding.

## Highlights

• The IFAC proposes seven types of frontoethmoidal cells.
• This study is the first to evaluate prevalence of cells according to IFAC in Brazil.
• This classification has a high Intraclass Correlation Coefficient.
• Agger nasi cell was the most prevalent cell in this study.
• Supra bulla frontal cell was the least prevalent cell.

## Introduction

Endoscopic frontal sinus surgery is steel a challenge,^1^ due to its complex anatomy, including the possibility of pneumatization in different cells, generating a huge variation in anatomical structures. In addition, the proximity to noble structures such as the cribriform plate, orbit, and anterior ethmoidal artery can increase this difficult.^2^

However, endoscopic approaches to the frontal sinus have grown significantly in the last decades^3^ reinforcing more and more the importance of adequate anatomy study for surgeons, in addition to the need for detailed preoperative preparation with imaging exams^4^ minimizing procedure related risks and providing a proper frontal sinus dissection, achieving better postoperative results.^5^

Several anatomical classification methods have already been proposed for frontal sinus, however, these previous systems present limitations of anatomical details, in addition to interobserver subjectivity.^4^ In 2016, the International Frontal Sinus Anatomy Classification (IFAC) was described by Wormald et al.^6^ This classification proposes seven types of frontoethmoidal cells based on their anatomical positions and relationships, allowing a more precise nomenclature, and facilitating communication between surgeons to promote a better description of surgical techniques in the teaching process, in addition to greater precision in surgical planning.^6^ The IFAC cells description can be seen in Table 1.

**Table 1:** International Frontal Sinus Anatomy Classification (IFAC).

Cell type	Cell Name	Definition	Abbreviation
Anterior cells (push the drainage pathway of the frontal sinus medial, posterior or posteromedially)	Agger nasi cell	Cell that sits either anterior to the origin of the middle turbinate or sits directly above the most anterior insertion of the middle turbinate into the lateral nasal wall	ANC
	Supra agger cell	Anterior-lateral ethmoidal cell, located above the agger nasi cell (not pneumatizing into the frontal sinus)	SAC
	Supra agger frontal cell	Anterior-lateral ethmoidal cell that extends into the frontal sinus	SAFC
Posterior cells (push the drainage pathway anteriorly)	Supra bulla cell	Cell above the bulla ethmoidalis that does not enter the frontal sinus.	SBC
	Supra bulla frontal cell	Cell that originates in the supra-bulla region and pneumatizes along the skull base into the posterior region of the frontal sinus	SBFC
	Supraorbital ethmoid cell	An anterior ethmoid cell that pneumatizes around, anterior to, or posterior to the anterior ethmoidal artery over the roof of the orbit.	SOEC
Medial cells (push the drainage pathway laterally	Frontal septal cell	Medially based cell of the anterior ethmoid or the inferior frontal sinus, attached to or located in the interfrontal sinus septum	FSC

Adapted from Wormald PJ et al., The International Frontal Sinus Anatomy Classification (IFAC) and Classification of the Extent of Endoscopic Frontal Sinus Surgery (EFSS). Int Forum Allergy Rhinol. 2016 Jul; 6(7):677-96. doi: 10.1002/alr.21738. Epub 2016 Mar 14. PMID: 26991922.

The aim of this study was to stablish the prevalence of each type of frontal cell according to IFAC, in the population of a Brazilian tertiary hospital, without sinus disease historic, trough not contrasted tomography study of the sinuses, in addition to observing the reliability of the classification between observers.

## Methods

This was an analytical cross-sectional study. We evaluated 103 computerized tomography exams of the sinuses, present in the image database of the Hospital de Clínicas of the State University of Campinas, performed between January 2020 and June 2021. The exams were randomly selected, respecting inclusion and exclusion criteria. Sample size was defined based on previous studies.

For inclusion criteria, tests performed on patients over 18 years old, computed tomography scans with thin sections, under 0.3 mm, which allowed reconstruction in axial, sagittal and coronal sections, and exams performed without contrast. We excluded from the analysis exams of patients with previous endonasal surgery, presence of craniofacial genetic abnormalities or a history of facial trauma. Also, patients with chronic rhinosinusitis were excluded because the aim of this study was to evaluate the overall population prevalence without the influence of inflammatory process that could modify anatomy. In addition, the absence of sinusitis makes it easier to identify the boundaries of the cells.

Each exam was independently evaluated by three experienced researchers (two radiologists and an otolaryngologist with training in rhinology), and the images were analyzed in triplanar viewer: coronal, sagittal, and axial sections, using PACS Arya® version 20.4.0, including simultaneous analysis for better identification of the cells.

The prevalence of each type of frontal cell was evaluated, according to International Frontal Anatomy Classification. Each researcher analyzed the same exams individually, each side at a time, and was blinded to their colleague's assessment. We also established de agreement between the evaluators to identify the cells, through the Intraclass Correlation Coefficient (ICC). For classifying reliability, we considered ICC < 0.40 poor reliability, 0.40‒0.59 moderate reliability; 0.60‒0.74: good reliability, and ICC 0.75–1.0: excellent reliability.^7^

For purposes of prevalence, the correct classification of frontal cells was considered when there was an agreement between the three examiners, or between two examiners. In cases of disagreement among the three examiners, a board composed of a radiologist and a senior otolaryngologist made the final assessment.

Data were processed with SPSS 16.0 software (SPSS Inc., Chicago, IL, USA). The mean, standard deviation, median and extreme values of age were demonstrated using descriptive statistics. Interrater reliability among the examinators was assessed by measuring the Intraclass Correlation Coefficient (ICC) for each cell type.

## Results

One hundred and three exams were evaluated, 206 tomography sides in our selected cases. The population included 56 female and 47 male patients, the mean age was 48 years old, as shown in Table 2. Also, three sides had aplasia of the frontal sinus.

**Table 2:** Age of participants.

	Mean	Median	Min‒Max	SD
Age	48	44	18‒88	17.3

Table 3 shows each cell prevalence and ICC. Interrater reliability was “excellent reliability” for all cells evaluated. The most prevalent cell was Agger Nasi cell, followed by supra bulla cell in second place. The least prevalent was supra bulla frontal cell.

**Table 3:** Cell prevalence and intraclass coefficient.

	Prevalence	Intra Class coefficient (ICC)
Agger nasi cell (ANC)	95.63% (197 sides of 206)	0.940 (0.925‒0.953)
Supra agger cell (SAC)	37.86% (78 sides of 206)	0.866 (0.830‒0.896)
Supra agger frontal cell (SAFC)	37.37% (77 sides of 206)	0.884(0.854‒0.909)
Supra bulla cell (SBC)	77.18% (159 sides)	0.782 (0.685‒0.846)
Supra bulla frontal cell (SBFC)	30.09% (62 sides of 206)	0.776 (0.709‒0.828)
Supraorbital ethmoid cell (SOEC)	32.03 % (66 sides of 206)	0.876 (0.842‒0.904)
Frontal septal cell (SFC)	33.49% (69 sides od 206)	0.901 (0.973‒0.924)

## Discussion

The International Frontal Sinus classification was described aiming to propose a better understanding of the anatomy of frontoethmoidal cells and its relations to frontal recess. In this classification, not only the number and position of cells is considered, but also how these cells affect frontal sinus drainage.^6^ One of the mains purposes of this classifications is to facilitate the communication between the health team and the learning process among nasosinusal surgeons.^8^ Our study showed high reproducibility for the identification of the cells among reviewers, as all cells were associated with “excellent reliability”. This high interrater reliability, however, is applicable to our sample of individuals without sinus diseases, there is no available data to support this finding in patients with sinus diseases.

This study aimed to assess the prevalence of frontoethmoidal cells based in IFAC classification, first of this kind performed in a Brazilian population and carried out in the most populous state in the country.^9^ As a country with a large ethnic variety,^10^ studying the prevalence of frontal cells in our specific population in essential, as it could vary from previous studies based in European, Asian, and North American populations.

**Figure 1:**
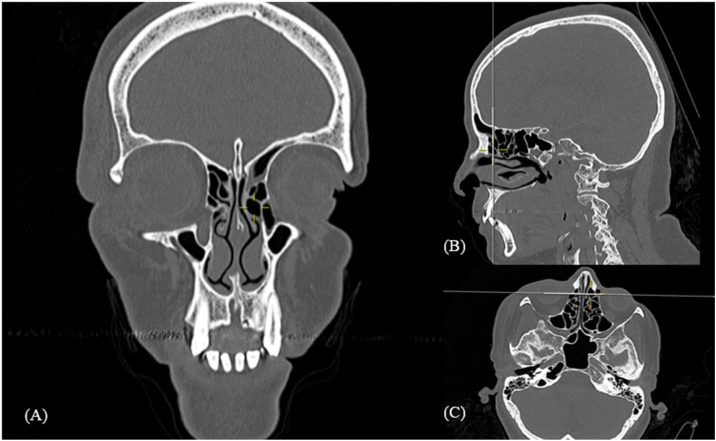
Agger nasi cell. Computed tomography: Mark shows left side agger nasi cell in coronal (A), sagittal (B) and axial (C) reconstruction.

When analyzing the results of cell prevalence, the agger nasi cell (Fig. 1) is the most prevalent one. Similar results were found in several other studies across the word.^4^, ^11^ This cell can be postulated as an important anatomic landmark, in preoperative planning during radiological analyses, and surgical procedures. This is due both its high prevalence in all populations already studied for its ease identification, as the most anterior ethmoidal cell, locates above the insertion of middle turbinate in the lateral nasal wall.^6^

**Figure 2:**
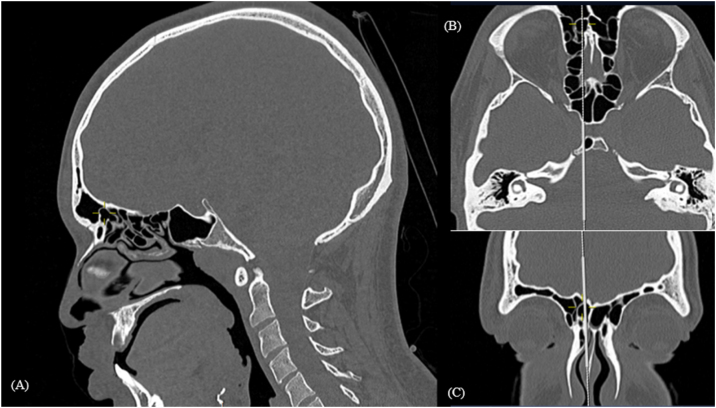
Supra agger frontal cell. Computed tomography: mark shows right supra agger frontal cell in sagittal (A), axial (B) and coronal (C) reconstructions.

**Figure 3:**
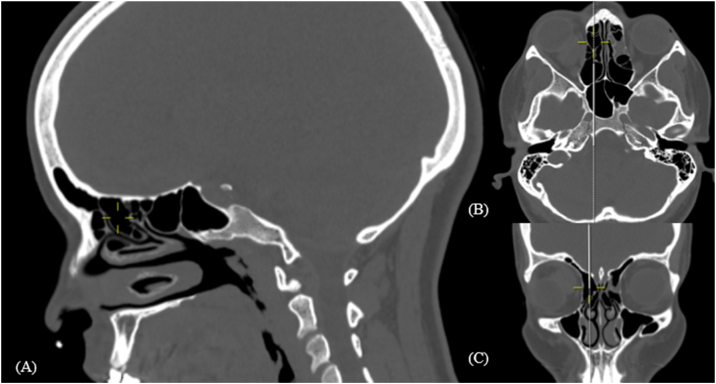
Supra bulla frontal cell. Computed Tomography: mark shows right supra bullar frontal cell in sargital (A), axial (B) and coronal (C) reconstructions.

Despite the homogeneity in high prevalence of agger nasi cells, when comparing all other cells prevalence, we notice a wide variability between the studies, especially when comparing prevalence of cells that insinuate in the frontal sinus, by the IFAC definition: supra agger frontal cell (Fig. 2) and supra bulla frontal cell (Fig. 3). As seen in a 7.88% SAFC prevalence found in Mexican study by Bravo-Arteaga, et al.^12^ versus a 37.37% prevalence of this cell in our prevalence study. Similarly, SBFC had a 4.3% prevalence in study performed in Vietnam by Luan V. Tran^13^ while 53% prevalence of SBFC was found in a study from Malaysia.^14^ A possible confounding factor related to those cells’ classification occurs because when analyzing parasagittal sections, often the height of the cell is visualized exactly at the level of frontal ostium, making it unclear if the cell advances in the frontal ostium entering the frontal sinus.

**Figure 4:**
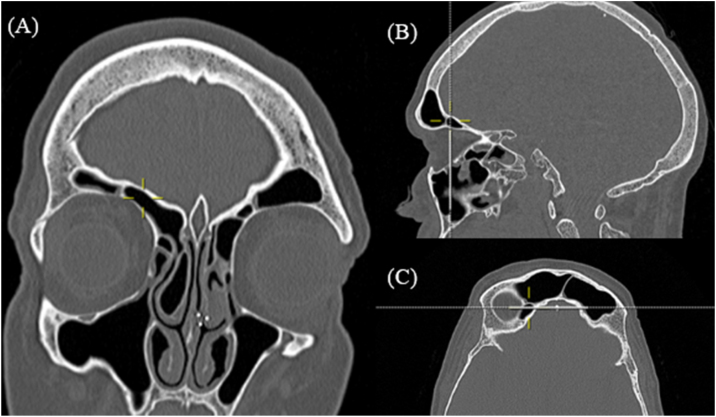
Supraorbital ethmoidal cell. Computed Tomography: yellow mark shows right supra supraorbital ethmoidal cell in coronal (A), sargital (B) and axial(C) reconstructions.

The Supraorbital Ethmoidal Cell (SOEC) is also found in a heterogenic prevalence among the studies. Previous Asian prevalence analyses, such as Chinese study by Zhang et al.^15^ found a small prevalence of 5.4% of SOEC, and a study by Cho et al.^16^ comparing the Korean population and Caucasian population regarding the prevalence of frontal cells, found SOEC (Fig. 4) less common in a Korean population.

## Conclusion

This study describes the frontal cell prevalence in a tertiary hospital in Brazil, using the IFAC. Agger nasi cell was the most prevalent cell in the study, followed by supra bulla cell as the second most prevalent. The least prevalent cell was the supra bulla frontal cell. The prevalence of the agger nasi cell is very similar among studies, however when comparing the prevalence of cells that insinuate in the frontal sinus, there is high variability in literature.

The IFAC proved to be a useful tool for evaluating anatomical variability of the frontoethmoidal cells, and it has proven reliable among examinators.

## Authors’ contributions

REFCDB drafted the article, participated in data collection, and revised the literature, MDCT was responsible for the conception of the article, analysis of the prevalence of frontal cells, statistical analysis, and correcting the final version of the article, ETIS and VSBM was responsible for evaluation of frontal cells prevalence, ES was responsible for the conception of the study and correcting the final version of the article.

## Conflicts of interest

The authors declare no conflicts of interest.
